# Building Connections with Families: Implementation of a Video-Messaging Service in the Neonatal Intensive Care Unit

**DOI:** 10.3390/children10081338

**Published:** 2023-08-02

**Authors:** Stephanie Bott, Nicole Dantas Fernandez, Janet Narciso, Janet MacAlpine, Nicole Quain, Julia Rettie, Lia Sharpe, Yenge Diambomba, Ayah Al Bizri, Karel O’Brien, Vibhuti Shah

**Affiliations:** 1 Department of Nursing, Mount Sinai Hospital, 600 University Avenue, Toronto, ON M5G 1X5, Canada; stephanie.bott@sinaihealth.ca (S.B.); nicole.dantasfernandez@sinaihealth.ca (N.D.F.); janet.narciso@sinaihealth.ca (J.N.); janet.macalpine@sinaihealth.ca (J.M.); 2 Department of Paediatrics, Mount Sinai Hospital, 600 University Avenue, Toronto, ON M5G 1X5, Canada; nicolequain@aol.com (N.Q.); julia.holt18@gmail.com (J.R.); liasharpe@gmail.com (L.S.); yenge.diambomba@sinaihealth.ca (Y.D.); ayah.albizri@sinaihealth.ca (A.A.B.); vibhuti.shah@sinaihealth.ca (V.S.)

**Keywords:** video-messaging service, family integrated care, neonatal intensive care

## Abstract

Background: Family involvement is vital to optimize the care of infants in the neonatal intensive care unit (NICU). Various technologies have been used to support communication with parents in the NICU. The purpose of this study was to evaluate the parent and staff experience and perception of the use of a cloud-based video-messaging service in our NICU. Methods: This study was a single center observational study conducted at Mount Sinai Hospital, Toronto, Canada. Following the implementation of a video-messaging service, parent and staff surveys were distributed to evaluate their experience and perception. Results: Parent responses were positive with respect to how the service helped them feel: closer to their infant (100%) and reassured about their infant’s care (100%). Nursing staff responses indicated that they perceived a benefit to parents (100%) and to their building a relationship with families (79%). However, they also identified time constraints (85%) and the use of the technology hardware (24%) as challenges. Conclusions: The use of an asynchronous video-messaging service was perceived as beneficial to both parents and staff in the NICU. Complaints pertained to the impact of the technology on nursing workflow and the difficulty using the hardware provided for use of the service.

## 1. Introduction

Admission of an infant to the Neonatal Intensive Care unit (NICU) after birth causes significant stress for parents and families due to infant separation as well the need to acclimatize to the intensive medical environment [1,2]. Family integrated care (FICare) has been implemented within NICUs around the world and has been shown to decrease parental anxiety and stress and increase exclusive breastfeeding and infant weight gain while promoting parent–infant bonding [3]. Within this model of care, parents are encouraged to be present at the infant’s bedside as much as they are able and participate in infant care activities including performing skin-to-skin and participating in medical rounds [4].

The COVID-19 pandemic presented new challenges within the world of healthcare for several reasons. Specifically within NICUs, parental presence was restricted in most hospitals. Prior to the pandemic, at Mount Sinai Hospital (MSH) NICU, parents were recognized as essential care-providers and could visit the NICU 24/7. Siblings and extended family were also able to visit regularly. With the onset of the COVID-19 pandemic, the caregiver policy was changed to allow only one parent to be present with their infant in a 24 h period. Parents in the NICU are at an increased risk of anxiety, depression, and post-traumatic stress disorder (PTSD), and unfortunately, the COVID-19 pandemic exacerbated these negative outcomes [5]. To communicate with families who were unable to be present during this time period, our unit increased the use of Zoom^®^ interface to include parents on medical rounds daily (Monday–Friday).

Neonatal intensive care units around the world have implemented various technological strategies to promote the engagement of parents in their infant’s care. These technologies include the use of webcams providing 24/7 live streaming of their infant, web-based applications that allow for the sharing of pictures and videos, virtual family centered rounds, and technology to provide parent education and support. At the time this study was designed, there was emerging research in this area that suggested that the use of these technologies may have a positive impact on parents [6,7,8,9].

In response to the restrictions placed around presence of families, a team in our NICU sought to introduce and evaluate the use of a secure web-based asynchronous video-messaging service (service) for our parents. This technology enables bedside nurses and unit volunteers to record pictures/videos of infants and send them directly to parents. At the time of study conception, there was no published literature on the use of this technology in the NICU. The purpose of this study was to evaluate the parent and staff experience and perception of the use of a video-messaging service in our NICU.

## 2. Materials and Methods

### 2.1. Setting and Study Design

This study was a single center observational study conducted at MSH NICU. The MSH NICU is a 62-bed single-room design tertiary care NICU, four of which can accommodate twins and have internal connecting doors for greater number of multiples. Nursing staffing ratios are typically 1 registered nurse: 2 babies. The NICU is a locked unit, and parents enter the unit using a biometric scanner at the entrance; parents have access to their baby’s bedside 24/7. The study was conducted over three phases: (1) the Pre-implementation phase (1 June to 31 December 2020), (2) the Implementation phase (1 January 2021 to 28 February 2022), and (3) the Evaluation phase from 1 April to 31 October 2022.

### 2.2. Pre-Implementation Phase

In order to support the implementation of the cloud-based video messaging service (vCreate^®^, vCreate Ltd., Windsor, UK), a steering committee and an operational team were formed. The steering committee was composed of three veteran parents, NICU leadership (medicine and nursing), staff physicians, a parent resource nurse, and a social worker. This team had the responsibility of overseeing the project and ensuring that the environmental conditions in the NICU would facilitate the introduction of the service. These conditions included staff engagement and support, corporate agreements to support the service, and support for staff to work on the implementation and evaluation. The operational team consisted of healthcare professionals (physicians, nurses, social worker, and three veteran parents) and was responsible for all activities at the unit level including staff and family orientation and training of both the hardware and software.

As part of the pre-implementation phase, the operations team distributed a cross-sectional survey to the bedside nurses to collect information regarding the feasibility of using this service and any anticipated barriers. Fifty-five responses were received. Sixty-six percent of respondents believed that the proposed technology would be a positive addition to our NICU. However, concerns were raised about expectations from parents for frequent photos/videos and the nursing ability to provide more than 1–3 photos/videos a week given the acuity of our unit. These findings led to the creation of a guidelines for the use of the video-messaging service, and adjustments were made to information given to parents to ensure that their expectations could be met. The developers of the video-messaging service trained five champions (nurses) to conduct the staff training. Other key decisions that were made included: (1) the choice of equipment for staff to use/interact with the service, (2) the frequency of photos/videos that could be sent to the families, (3) the process for providing information and registering parents within the service, (4) assuring that the participant consent was obtained through the service, and (5) how to provide staff education about the service. The process for enrolment of families is as follows: parents were approached by one of the NICU team members or saw information on posters in the NICU. If interested, parents received two emails: one email providing information on the frequency of photos/videos they could potentially receive and the second email for them to register through the service. The steering committee approved the purchase of a license to use the video-messaging service, and tablets were chosen as the interface for use in the NICU.

### 2.3. Implementation Phase

The hospital approved the use of service in December 2020. Each infant whose family was enrolled received a QR code to confirm their participation and was linked to the parents’ email address where photo/videos could be sent. The nurses used the service web app on the tablets to take and send photos or short 1–3 min videos to the family account. Families could access the photos/videos by logging into their account and had the options to view, download, or share the photos and videos with other family members. Data privacy and protection were ensured by using a National Health Service Digital Library app (video-messaging service), which provided secure cloud data storage (Microsoft Azure, Microsoft, Washington, DC, USA) for the images/videos and secure access to parents through a password protected login interface. Moreover, the NICU’s single family room design eliminated the risk of recording other infants by accident.

Between 1 January 2021 and 31 March 2021, we first piloted the service on 11 families who received 110 photos/videos. Lessons learnt from this pilot phase led to changes in the procedures in response to staff and parent feedback. For example, challenges were identified with the scanning of the QR codes. This was resolved by changing the placement and lamination of the label and ensuring that there was sufficient light that made it easier to scan while still maintaining infection control practices. A sticker was also placed in the infant’s room so that nurses could document when the last photo/video was taken. Laminated signage was placed on the door of participating infant which indicated parents’ consent to send them photos/videos. Parents were concerned that some of the pictures were blurry, and light was adjusted in the room to ensure that the quality of the photos/videos they received were optimal.

Concurrently, all staff nurses were trained, and an additional 13 champions were identified. The use of the service was fully implemented on 1 April 2021. As the COVID-19 restrictions were modified, volunteers were reintroduced into the NICU on 1 September 2021, and they provided support to the nurses in using the service and taking photos/videos.

### 2.4. Evaluation

Parents and nurses/volunteer surveys were used to assess the parent and staff experience of the use of the video-messaging service on the following aspects: technological aspects (e.g., ease of use, quality of images, the ability to share images with parents and families, and concerns of privacy) and parents’ perceptions on attachment, breastmilk pumping, stress and delivery of care, and connection to bedside nurses. Similarly, nurses’ experience with the technological aspects of the service (ease of use, availability of tablets to obtain photos/videos, training and support provided, and concerns regarding privacy), relationship with parents, and workload and time constraints during nurses’ shifts were evaluated. The responses to the questions were provided using a Likert scale ranging from completely disagree to completely agree. A column labeled “Non-applicable” was also included if needed. In addition, a free text box was available for parents and nurses/volunteers to provide comments and feedback that was not captured in the survey questions. Novi Survey© (Cambridge, MA, USA), a user-friendly online survey tool, was used for distributing the parent and staff surveys. The surveys were distributed to parents who were enrolled in the program via email, and nurse/volunteer surveys were distributed via hospital group emails from 1 April 2022 to 31 October 2022. The survey tools were approved by the MSH Research Ethics Board.

### 2.5. Data Variables

In addition to the surveys, demographic data were collected for both groups. For parents, these variables included the parent who completed the survey, age, marital status, level of education, ethnicity, and gestational age at birth. Demographic data for nurses/volunteers included years of employment at MSH, nursing experience in years, level of education, and age.

### 2.6. Sample Size Calculation

The video-messaging service was introduced in our unit during the COVID-19 pandemic for the first time, and recognizing that this was a feasibility study, no sample size calculation was performed.

### 2.7. Outcome Measures

Outcomes for both groups were categorized as follows: technological aspects (ease of use of the service, need for training and support, barriers to the use of service, concerns regarding the number of photos/videos sent/received, and concerns regarding privacy). Parental perceptions including attachment, breastmilk pumping, parent–staff relationship, and parental anxiety and frustration were evaluated. Nursing/volunteer perceptions included relationship with families, training and support on the use of the service, workload issues, and time constraints were evaluated.

### 2.8. Statistical Analysis

Data analysis was conducted using Microsoft Excel 2013. Results are presented as numbers (percentages) as appropriate. Logistic regression analysis was conducted to study the association between years of experience as a nurse and the video-messaging service survey with a statistical significance set at *p* < 0.05. Feedback from both groups is also presented.

## 3. Results

### 3.1. Family Survey Results

From 1 January 2021 to 28 February 2022, 152 families expressed interest in participating in the program. Of these, 19 were parents of multiples (16 sets of twins and 3 sets of triplets). One survey per family was distributed to each family. Of the 152 families, responses were provided by 54 of them (35.5%). Surveys from eight families were excluded from this evaluation as one did not receive an email to register, one received an email but never registered, and six families registered but never used the service. Hence, only 46 of the 54 (85%) families downloaded and used the service. Of the respondents, 78% of the surveys were completed by the mothers. Eighty-three percent of the responders were living with a partner/spouse; seventy-four percent were in the age group of 31–40 years; and seventy-four percent had at least college degree. Eighty-five percent of the infants born to these families were <30 weeks gestational age at birth.

Families’ responses to their experience and perception of video-messaging service are presented in Table 1. Families experienced increased attachment, felt happier and closer to their infant, and felt reassured that their infant was receiving appropriate care (100%). Mothers expressed that it was easier to pump milk at home after seeing the photos/videos (73%). The process of registering and downloading the app was easy; the resolution of the photos/videos were of good quality; and they were able to share them with extended family members. Twenty-six percent of families were frustrated with the staff when they did not receive any photos/videos, and they wanted videos of longer duration. Overall, 100% of the families stated that they would recommend the use of this app.

Positive and negative comments from the parents included (additions in brackets to explain quotes): (1) “We loved this! The primary nurses were the best at sending photos/videos! We didn’t get much if his primaries (primary nurses) weren’t with him and it would have been nice to see photos/videos from nurse(s) we did not know to make us more comfortable”; (2) “vCreate was amazing and helped me see the great care and bonds my twins were receiving/benefiting from when I couldn’t be there”; (3) “The application would not allow me to send videos which is fine. It let me send the photos to family members. I think it’s an amazing idea! I was so far away from the hospital it helped a lot!”; (4) “The picture was a little blurry at times”; and (5) “I was registered, but I never got any photos or videos”.

### 3.2. Nursing Survey Results

From 1 April 2022 to 31 October 2022, the survey was distributed to all nursing staff (*n* = 196) of which 115 completed the survey. Six responses were excluded as the nurses indicated that they had never used the service. Of the remaining 109 nurses, 35% had <4 years of experience as a nurse, and 46% had <4 years of experience at MSH. Forty-three percent of the nurses were in the age group of 20–29 years, and 84% had a Bachelor’s degree. Half of the nurses reported having used the service < or equal to ten times.

Nurses’ responses to their experience and perception of video-messaging service are presented in Table 2. Ninety-five percent of staff indicated that they liked being able to send pictures/videos to parents, and all indicated that they felt sending photos/videos to parents’ reduced parental stress while their baby is in the NICU and fostered attachment with their infant. Even though the nurses felt that sending photos/videos helped them feel satisfied about their workday and were more connected to the infant and parent, 29% felt pressure from families to send them photos/videos. While the nurses felt supported by app champions (76%), a third found it hard to learn how to use the app, and around 25% had technical difficulties with app interface, uploading errors for videos, and use of the tablet stand. A third of the nurses reported needing more training to use the app.

Logistic regression showed that nurses’ responses were associated with their years of experience. Nurses with >10 years of experience were more likely to report technological challenges with the use of the video-messaging service (odds ratio (OR) 2.83, 95% confidence interval (CI) 1.04, 7.74) and the need for more training to use the video-messaging service (OR 14.76, 95% CI: 4.55, 47.94) as compared to nurses with 4–10 years of experience (reference category).

Positive and negative comments from the nurses included (additions in brackets to explain quotes): (1) “vCreate is a great tool and parents are very appreciative of the updates they receive from the application. However, my main issue is the iPad itself. It is very bulky and I find it very difficult to wheel to the pt. (patient’s) room. I have also have (needed) to physically lift the iPad (and its stand) to take photos for babies in cribs. I have to lift the iPad and stand very high to take photos. Getting hit by the (stand) wheel is not pleasant”; (2) “I actually think it makes it more like a little playtime with the baby and therefore it is fun and rewarding to take part in”; (3) “Processing the video clips take time and occasionally it comes up as a processing error initially- but after waiting for 5–10 min the video appears. This time delay is a reason I usually take photos instead of videos”; (4) “It feels like another task to check off on my to do list”; and (5) “need more practice”. Several nurses commented that this task should be left for volunteers.

### 3.3. Volunteer Survey Results

Seven survey responders indicated that they were volunteers. All had <3 years of experience volunteering, and 85% of them had a high school diploma, were <20 years of age, and had used the app > than 21 times. Volunteers’ responses to their experience and perception of video-messaging service are presented in Table 3. All volunteers liked being able to send photos/videos to parents, indicating that sending photos/videos to parents reduced parental stress while their baby is in the NICU. They felt that sending photos/videos helped them feel better about their workday and more connected to baby and parent. They reported no difficulties in using the app and indicated that they needed no further training.

Volunteers commented that “vCreate is amazing for both us volunteers, as well as families of the NICU babies! It is essential and so helpful” and “Feedback I hear from parents is how much of a positive difference it makes for them”.

## 4. Discussion

### 4.1. The Results of the Parent and Staff Evaluation of the Impact of Video-Messaging

In this study, families indicated that the use of the service appeared to facilitate both parent–infant attachment and parent–staff closeness. Parents reported an emotional closeness to their child and less anxiety when separated, as well as greater trust in the care of their nurse when using the service. Staff also reported that they felt that their relationships with families were strengthened by using the service. Building a partnership between parents and staff is a key component of Family Integrated Care, so it was very important that this technology supported this. In addition, the staff responses in the nursing survey suggested that using this technology could make a nurse feel better about their workday. This may merit further exploration as improved job satisfaction could mitigate the increasing risk of staff burnout. Unfortunately, sending parents photos and videos is time-consuming. Staff reported time constraints as a barrier to this task. Volunteers were brought in to assist nurses and appear to have been successfully doing so, sending >21 videos/pictures, and as a result, staff may have missed out on the opportunity to develop a closer relationship to parents by sending these photos/videos. Despite staff concerns that they might not be able to meet the expectation of families, it appears that parents are very satisfied even when they received limited photos/videos. This may be in part due to the clear messaging that was provided to parents when they signed up for the use of the service and also an appreciation of the work that the staff are doing.

### 4.2. The Results of the Parent and Staff Evaluation of the Implementation of Video-Messaging

The results of the parent survey indicate that the implementation of this technology appeared to be well facilitated for parents. However, the nurses’ surveys indicate that there were many challenges with implementation of the service in our unit. Additional infection control safety precautions were in place at the time of implementation, necessitating the tablets to remain on specific stands that did not come into contact with anything close to the baby. In addition, as the tablets were expensive to replace they were tethered to a stand so they could not be removed from the unit. The nurses’ surveys clearly indicate that the placement of the tablets was a barrier to them being able to take videos easily and at a suitable time for them. This likely contributes to their perception of increased workload. In addition, although the implementation team had felt that they planned the implementation in a way that would support uptake in the unit, it appears that the staff did not feel well listened to, and almost a third indicated that they required more support with using the technology.

### 4.3. How Do Our Results Compare to Other Published Data?

An evaluation of the same asynchronous video-messaging service was reported by the team from Glasgow in 2021. It is significant that our findings were similar to theirs in that video-messaging was demonstrated to improve the parents experience in the NICU and to build the relationship with parents and staff. However, in contrast to our results, their staff indicated that the technology was easy to use, and their major concern was managing parents expectation in relation to the number of videos that could be sent [8].

Many studies have highlighted the challenges of using technology to support communication with parents of infant in the NICU [6,7,9]. Several different technological interventions have been proposed to improve communication and to promote interactions among NICU staff and parents including videoconferencing, (Skype^®^, Facetime^®^, and Zoom^®^) and commercial modalities such as different webcams as well as video-messaging systems. Although many of these interventions have been shown to improve staff–parent communication, an integrative review published in 2016 and a recent scoping review in 2023 suggest that further research is still required specifically to understand the experiences of nursing staff [9,10]. A report by Joshi et al. evaluating the impact of webcams on nursing workload demonstrated that nurses spent a significant amount of time adjusting cameras and addressing parental concerns, causing disruption to their workflow. They came to the conclusion that careful attention needs to be paid to how technology is implemented in the NICU. These findings support those of our study, where the technology, may have had an impact on our nursing staff workflow. The outcome, although not measured directly in our study, was underuse of the technology which was then addressed by bringing in volunteers to help support our nursing staff with this task. Our nursing staff clearly identified concerns about the hardware being used for the service. Functionality and ease of use of the hardware are identified as being very important when considering how staff can efficiently use technology in the NICU. This has also been reported as an issue with bedside webcams [6,7]. As we have identified in our study, lack of confidence using the technology, the need to manipulate equipment, and troubleshooting technical issues have also been reported with the use of other video communication tools [7].

### 4.4. What Are the Strengths of This Study?

This is the first Canadian site to report on the use of an asynchronous neonatal video-messaging service with families in the NICU and its evaluation. A multidisciplinary team, including parents, was involved in planning the implementation and the evaluation of the service. The nursing staff and volunteer evaluation was completed a year following implementation, allowing users to become very familiar with the service before being asked to reflect upon its use.

### 4.5. What Are the Limitations of This Study?

A serious limitation of our study is that the parent survey response rate was low and the parent surveys were distributed to parents at varying times from their admission. We were unable to approach parents for consent while in hospital but emailed the survey to those who registered an interest in using the service over the first year of implementation (2021). This sample may therefore not be fully representative of the population of parents in our NICU and may also be prone to recall bias. That said, the collected parent demographics indicate that the surveys are from a very varied population of parents. Unfortunately, we also do not have data on the length of stay of the infant and family in our unit. We could therefore not assess how the length of stay compared to the number of pictures and videos that were received by each family.

This study reflects a specific snapshot evaluation of our implementation journey. We have seen that the use of the service appears to be increasing with time with the COVID-19 pandemic receding and our NICU staffing being more stable. The video messaging service, while not a replacement for parental presence in the NICU, provides support to parents when they cannot be present with their baby at some time during the day. Of note, there is a cost to this service. The cost is NICU-specific and is determined by the service provider based on bed numbers as a representation of likely usage and in many units has been supported by foundations or charities. Our study results provide further direction for sites anticipating using any technology to support parent interaction in the NICU. As our results indicate, it is critically important to understand if the technology works for all involved, both parents and staff. This study highlights the need to address staff concerns with respect to the ease of use of this technology as it is unlikely be sustained even if staff understand its potential benefits to families without first addressing those barriers.

## 5. Conclusions

The use of an asynchronous video-messaging service was perceived as beneficial to both parents and staff in the NICU at Mount Sinai Hospital. The largest area of complaint within the nursing survey pertained to the difficulty using the hardware provided for use of the video-messaging service and the negative impact of the technology on nursing workflow. Seeking ongoing feedback from the users, in our case bedside nurses, and understanding how they interact with the technology will enable us to improve the use of this tool over time.

## Figures and Tables

**Table 1:** Parents’ experiences and perceptions of using the video-messaging service (*n* = 46).

Survey Questions	Agree (%)	Disagree (%)
Receiving photos/videos of my baby made me feel closer to my baby	100	0
Receiving photos/videos of my baby made me more anxious at home	7.7	92.3
Seeing my baby’s photos/videos made me feel happier when at home	100	0
Seeing my baby’s photos/videos made it easier for me to pump milk	73.3	26.7
I liked being able to share the photos/videos with other family members	97.5	2.5
Receiving photos/videos helped me feel reassured about the care my baby was receiving	100	0
Receiving photos/videos made me feel more connected to my baby’s nurse	94.7	5.3
I am satisfied with the number of photos/videos that I received	80	20
I would have liked to have received longer video recordings	69.4	30.6
I was frustrated with staff when I did not receive photos/videos	26.3	73.7
It would be better for me to receive the photos/videos via text message rather than by email	37.1	62.9
I had difficulty accessing the photos/videos because of my limited data plan or my device	5.4	94.6
I received information about the app in the week after admission to the NICU	69.4	30.6
It was easy for me to download the app	97.2	2.8
It was easy for me to register to use the app	94.6	5.4
I had technical difficulties using the app	14.3	85.7
The resolution of the photos/videos I received was good	87.2	12.8
I was easily able to send photos/videos to other family members	88.9	11.1
I was easily able to download photos/videos	89.5	10.5
I was concerned that the photos/videos of my baby were not secure on the app	7.9	92.1
I recommend the use of this app	100	0

**Table 2:** Nurses’ experiences and perceptions of using the video-messaging service (*n* = 109).

Survey Questions	Agree (%)	Disagree (%)
I liked being able to send photos/videos to parents	95.4	4.6
I think that sending photos/videos to parents reduces their stress while their baby is in the NICU	100	0
I think that sending photos/videos to parents helps parents to feel closer to their infant	99.1	0.9
Sending photos/videos to parents has helped me to feel more connected to the baby	66.4	33.6
Sending photos/videos to parents made me feel better about my workday	81.7	18.3
I think that sending photos/videos to parents has helped me build a relationship with parents	79.2	20.8
I feel that having this app has created more difficulties for me dealing with families	11.4	88.6
I am constantly feeling under pressure from families to send photos/videos	28.6	71.4
I have wanted to send photos/videos but have not been able to because of time constraints	85	15
I have wanted to send photos/videos but have not been able to do so because of access to an iPad/problems with the technology	53.3	46.7
I feel that sending photos/videos to families is not a priority for me working in the NICU	51.9	48.1
I have concerns about the security of sending photos/videos to families using vCreate^®^	11.3	88.7
I feel that my voice was listened to when planning how best to use the app	52.3	47.7
I found it hard to learn how to use vCreate^®^	32.4	67.6
I have had difficulty scanning the baby’s barcode while using vCreate^®^	41	59
I have had other technical difficulties using vCreate^®^ (please describe below)	24.2	75.8
I needed more training to use vCreate^®^	34	66
I have spent time supporting parents in their use of the app	19.3	80.7
I have had difficulty finding an iPad to take the photo/video when I have wanted to do so	42.1	57.9
I felt well supported by the vCreate^®^ champions in the unit	76.1	23.9

**Table 3:** Volunteers’ experiences and perceptions of using the video-messaging service (*n* = 7).

Survey Questions	Agree (%)	Disagree (%)
I liked being able to send photos/videos to parents	100	0
I think that sending photos/videos to parents reduces their stress while their baby is in the NICU	100	0
I think that sending photos/videos to parents helps parents to feel closer to their infant	100	0
Sending photos/videos to parents has helped me to feel more connected to the baby	100	0
Sending photos/videos to parents made me feel better about my workday	100	0
I think that sending photos/videos to parents has helped me build a relationship with parents	100	0
I feel that having this app has created more difficulties for me dealing with families	0	100
I am constantly feeling under pressure from families to send photos/videos	0	100
I have wanted to send photos/videos but have not been able to because of time constraints	14.3	85.7
I have wanted to send photos/videos but have not been able to do so because of access to an iPad/problems with the technology	0	100
I feel that sending photos/videos to families is not a priority for me working in the NICU	0	100
I have concerns about the security of sending photos/videos to families using vCreate^®^	0	100
I feel that my voice was listened to when planning how best to use the app	50	50
I found it hard to learn how to use vCreate^®^	0	100
I have had difficulty scanning the baby’s barcode while using vCreate^®^	0	100
I have had other technical difficulties using vCreate^®^ (please describe below)	0	100
I needed more training to use vCreate^®^	0	100
I have spent time supporting parents in their use of the app	71.4	28.6
I have had difficulty finding an iPad to take the photo/video when I have wanted to do so	14.3	85.7
I felt well supported by the vCreate^®^ champions in the unit	100	0

## Data Availability

The data presented in this study are available on request from the corresponding author. The data are not publicly available due to privacy.
